# Calyxin Y sensitizes cisplatin-sensitive and resistant hepatocellular carcinoma cells to cisplatin through apoptotic and autophagic cell death via SCF βTrCP-mediated eEF2K degradation

**DOI:** 10.18632/oncotarget.19883

**Published:** 2017-08-03

**Authors:** Chao Zhang, Jian-Li Lei, Hao Zhang, Yuan-Zheng Xia, Pei Yu, Lei Yang, Ling-Yi Kong

**Affiliations:** ^1^ Jiangsu Key Laboratory of Bioactive Natural Product Research and State Key Laboratory of Natural Medicines, China Pharmaceutical University, Nanjing 210009, China

**Keywords:** calyxin Y, cisplatin, eEF2K, autophagic and apoptotic cell death, HCC

## Abstract

The down-regulation of eukaryotic elongation factor-2 kinase (eEF2K) is associated with an enhancement in the sensitivity of malignant cells to chemotherapeutic agents. In this study, we found that the silencing of eEF2K enhanced cisplatin (CDDP)-induced cytotoxicity in CDDP-sensitive (HepG2) and resistant (HepG2/CDDP) cells. Calyxin Y, a unique chalcone diarylheptanoid adduct, down-regulated eEF2K by promoting Skp1-Cul1-F-box protein (SCF) β-transducin repeat-containing protein (βTrCP)-mediated protein degradation and synergistically enhanced the cytotoxicity of CDDP. Subsequently, we identified a potential mechanism of this cooperative interaction by showing that the combination of calyxin Y and CDDP enhanced apoptotic cell death via mitochondrial dysfunction. In addition, the combination induced autophagy, which contributed to the synergistic cytotoxic effect. Further research revealed that calyxin Y synergistically sensitized HepG2 and HepG2/CDDP cells to CDDP through enhanced apoptotic and autophagic cell death via the SCF βTrCP-eEF2K pathway. Finally, *in vivo* studies demonstrated that calyxin Y could enhance the response of HepG2/CDDP cells to CDDP in xenograft models with low systemic toxicity. Thus, the combination of calyxin Y and CDDP might represent an attractive therapeutic strategy for the treatment of chemotherapy-sensitive and resistant hepatocellular carcinoma cells.

## INTRODUCTION

Hepatocellular carcinoma (HCC) is one of the most common malignancies worldwide, especially in East and South-East Asia and Northern and Western Africa [1]. Systematic chemotherapy plays a crucial role in HCC treatment, especially for patients with advanced HCC [2]. Cisplatin (CDDP) is commonly used as a chemotherapeutic agent for HCC, although it cannot satisfactorily improve the survival rate of patients with advanced HCC due to the reduced sensitivity that occurs with the development of drug resistance [3]. Revealing the cellular and molecular mechanism of the development of sensitization to chemotherapeutic agents is indispensable for the generation of effective HCC therapeutics.

Eukaryotic Elongation Factor 2 Kinase (eEF2K) is a unique Ca^2+^/calmodulin-dependent Ser/Thr kinase that regulates protein synthesis through the phosphorylation of eukaryotic elongation factor-2 (eEF2) [4]. This factor mediates GTP-dependent tRNA-mRNA duplex translocation through the ribosome. Therefore, its inhibition negatively impacts protein synthesis. In addition to the control of transcription, eEF2K expression is also inhibited through the ubiquitin-proteasome system. Such degradation requires the ubiquitin ligase SCF βTrCP (Skp1-Cul1-F-box protein, β-transducin repeat-containing protein) [5]. The eEF2K pathway restores cellular homeostasis during conditions of nutrient or energy depletion by decreasing translation rates at the stage of elongation. Within tumors, decreased protein synthesis by eEF2K activation has been proposed to contribute to tumor adaptation under nutrient depletion and stress conditions, including hypoxia and DNA damage [6].

eEF2K is overexpressed in different types of cancer, including HCC [7], and the expression correlates with poor patient survival. eEF2K regulates many cellular processes, such as protein synthesis, cell cycle progression, autophagy and apoptosis in cancer cells [8, 9]. In the past few years, eEF2K has been regarded as a potential cancer target. The down-regulation of eEF2K was associated with the enhancement of the sensitivity of malignant cells to chemotherapeutic agents. The inhibition of eEF2K has been reported to augment the effects of lapatinib on human nasopharyngeal carcinoma cells [10]. Moreover, silencing eEF2K sensitizes human glioma cells to TNF-related apoptosis-inducing ligand (TRAIL), temozolomide, and 2-deoxy-D-glucose (2-DG) [11-13]. Nevertheless, the role of eEF2K in the sensitivity of HCC cells to CDDP is still unclear.

Calyxin Y, a unique chalcone diarylheptanoid adduct, was isolated from *Alpinia katsumadai*. Calyxin Y exhibited significant growth inhibitory effects against several cancer cell lines [14]. Our previous study demonstrated that calyxin Y induced autophagy and apoptosis in non-small cell lung cancer cells [15]. In the current study, we established CDDP -resistant HepG2 (HepG2/CDDP) cells and investigated the role of eEF2K in the sensitivity of these cells to CDDP. Furthermore, we showed that a combination of calyxin Y and CDDP could synergistically inhibit cell viability and induce cell death in HepG2 and HepG2/CDDP cells. Finally, we identified a potential mechanism of this combination treatment that might be associated with the eEF2K pathway.

## RESULTS

### Silencing of eEF2K enhances CDDP-induced cytotoxicity in human HCC cells

**Figure 1:**
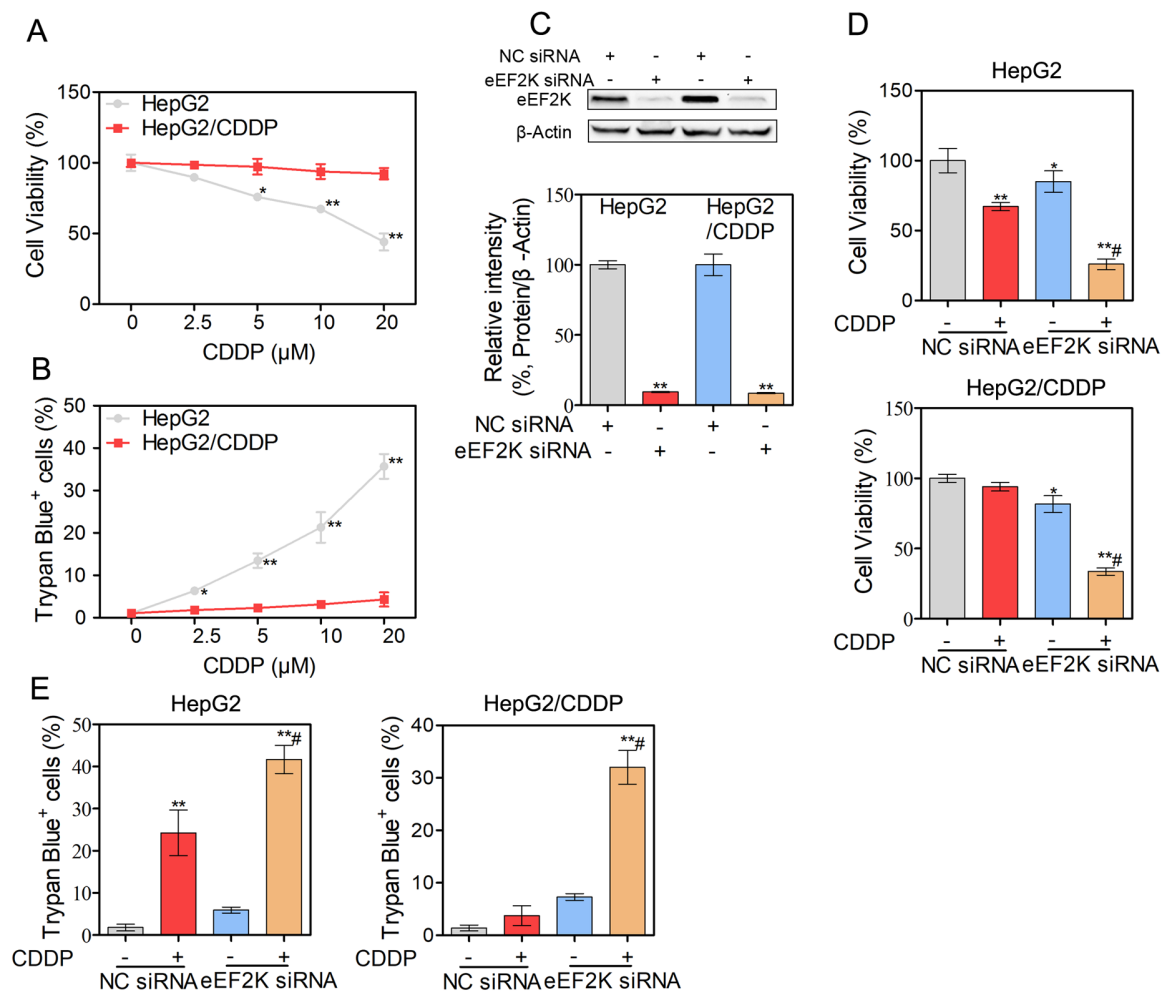
Silence eEF2K enhance CDDP-induced cytotoxicity in human HCC cells **(A-B)** HepG2 and HepG2/CDDP cells were treated with different concentrations of CDDP for 24 h. The cell viability was determined by MTT assay (A). Unattached and attached cells were collected and stained with trypan blue dye, and the numbers of dead cells were manually counted. The percentage of trypan blue^+^ cells represented the population of dead cells (B). **(C-E)** HepG2 and HepG2/CDDP cells were transfected with eEF2K siRNA (100 nM) or negative control (NC) siRNA. Twenty-four hours after transfection, the expression of eEF2K was measured by western blot. β-Actin served as a loading control (C). The cell viability was determined by MTT assay (D). Unattached and attached cells were collected and stained with trypan blue dye. The percentage of trypan blue^+^ cells represented the population of dead cells (E). Bars represent means ± S.D. of three independent experiments; *p < 0.05 and **p < 0.01, compared to non-treated control; ^#^p < 0.05, compared to CDDP treated NC siRNA transfected cells.

In this study, we first established CDDP-resistant HepG2/CDDP cells. The growth curve of HepG2 and HepG2/CDDP cells was shown in Supplementary Figure 1. HepG2/CDDP cells showed the lower proliferation rate compared with HepG2 cells. The difference between the wild-type cells (HepG2) and the resistant cells (HepG2/CDDP) was evaluated with the MTT and trypan blue exclusion assays. The cell viability in the HepG2/CDDP and HepG2 cells was comparable at approximately 2.5 μM of CDDP, after which point HepG2/CDDP cells were tolerant to CDDP (Figure 1A). CDDP induced a concentration-dependent increase in cell death in HepG2 cells. In contrast, the degree of cell death remained unchanged in HepG2/CDDP cells over the course of the culture (Figure 1B). The IC50 value of CDDP was 16.7 μM for HepG2 cells and 95.4 μM for HepG2/CDDP cells at 24 h, indicating that the resistance index was nearly 6-fold. Considering that eEF2K decreased the sensitivity of malignant cells to many agents [11-13], we wondered whether the suppression of eEF2K would increase the efficacy of CDDP in HepG2 and HepG2/CDDP cells. We knocked down eEF2K by siRNA and detected cell viability and cell death in response to CDDP exposure. The high knockdown efficiency of eEF2K in HepG2 and HepG2/CDDP cells was demonstrated by western blotting (Figure 1C). We found that both eEF2K-silenced HepG2 and HepG2/CDDP cells exhibited greater CDDP sensitivity than the control group (NC siRNA) (Figure 1D). In addition, silencing of eEF2K increased the death of HepG2 and HepG2/CDDP cells exposed to CDDP (Figure 1E).

### Calyxin Y exhibits cytotoxicity in human HCC cells

**Figure 2:**
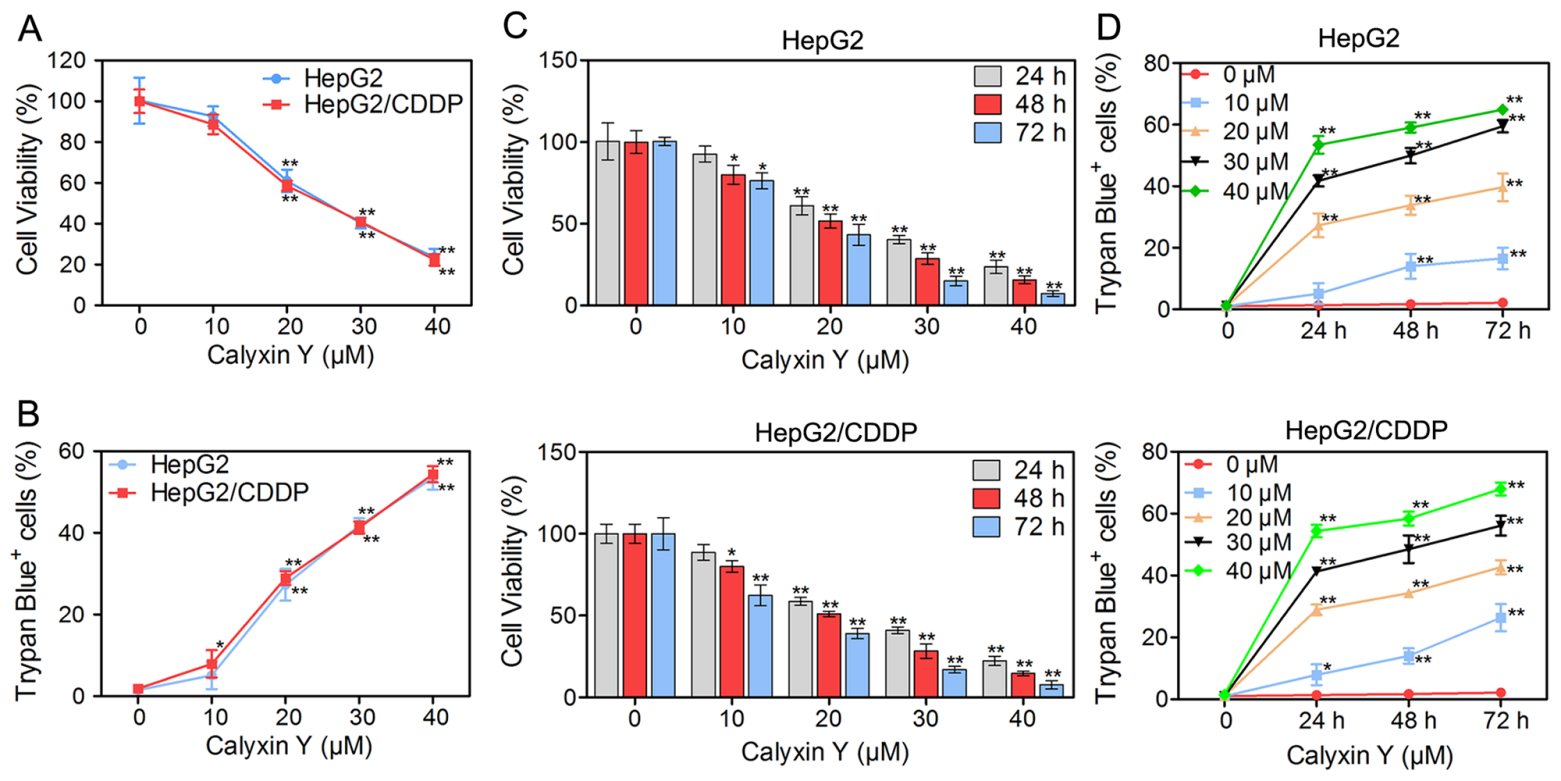
Calyxin Y exhibits cytotoxicity in human HCC cells **(A-B)** HepG2 and HepG2/CDDP cells were treated with various concentrations of calyxin Y (10, 20, 30, 40 µM) for 24 h. The cell viability was determined by MTT assay (A). Unattached and attached cells were collected and stained with trypan blue dye. The percentage of trypan blue^+^ cells represented the population of dead cells (B). **(C-D)** HepG2 and HepG2/CDDP cells were treated with various concentrations of calyxin Y (10, 20, 30, 40 µM) for 24, 48 and 72 h. The cell viability was determined by MTT assay (C). Unattached and attached cells were collected and stained with trypan blue dye. The percentage of trypan blue^+^ cells represented the population of dead cells (D). Bars represent means ± S.D. of three independent experiments; *p < 0.05 and **p < 0.01, compared to non-treated control.

As calyxin Y exhibited cytotoxic activity in several cancer cell lines [15], we then investigated the effect of calyxin Y on HepG2 and HepG2/CDDP cells. Cells were treated with 10, 20, 30, and 40 µM calyxin Y, and cell viability was assessed via the MTT assay. As shown in Figure 2A, calyxin Y inhibited the viability of HepG2/CDDP cells as much as it did that of HepG2 cells in a concentration-dependent manner. Trypan blue exclusion assays were used to confirm the death-inducing effect of calyxin Y on these cells (Figure 2B). In addition, calyxin Y reduced the viability of HepG2 and HepG2/CDDP cells in a time-dependent manner (Figure 2C) and increased cell death (Figure 2D).

### Calyxin Y decreases the expression of eEF2K by promoting SCF βTrCP-mediated protein degradation in human HCC cells

**Figure 3:**
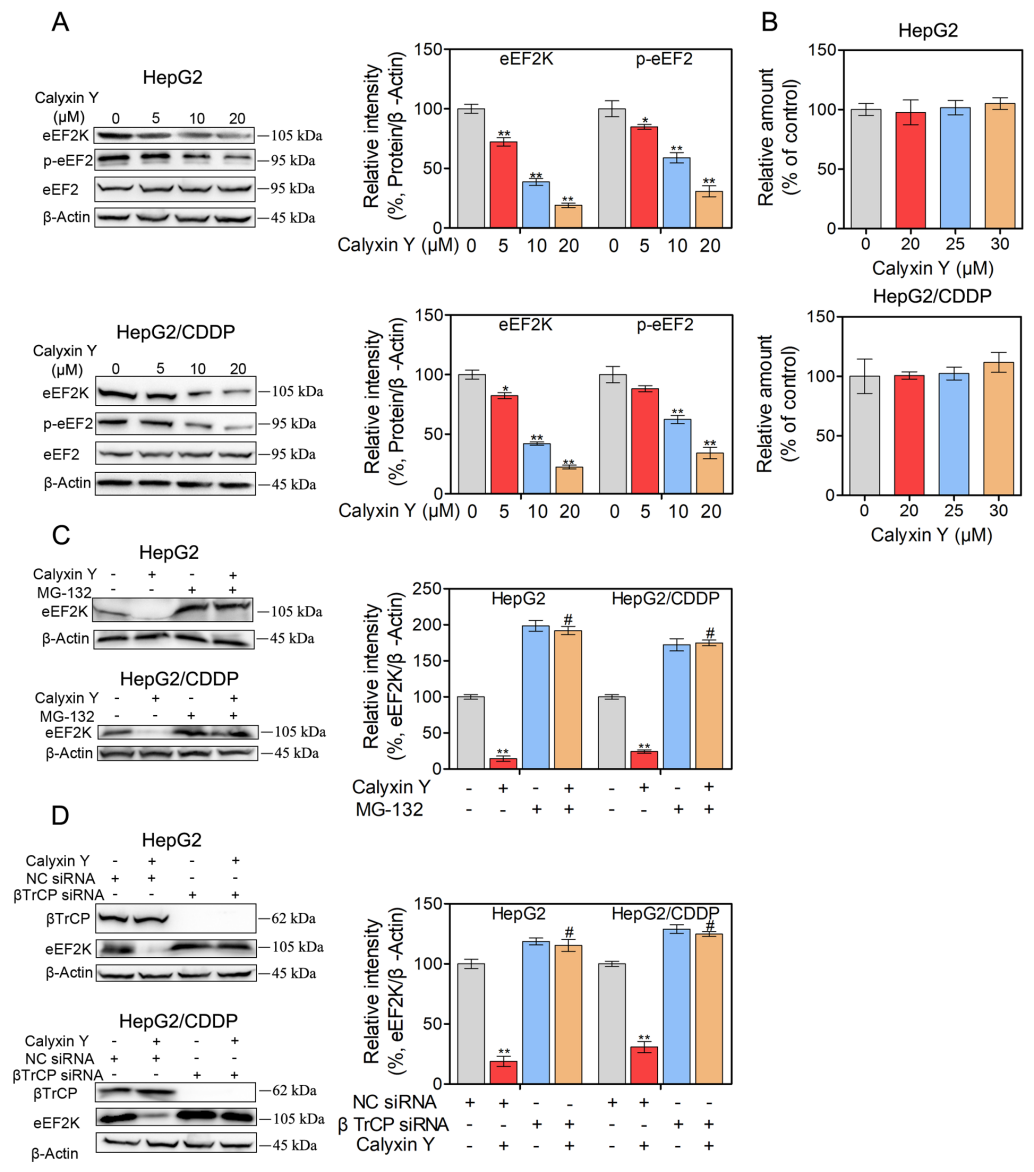
Calyxin Y decreases the expression of eEF2K though promoting SCF βTrCP-mediated protein degradation in human HCC cells **(A-B)** HepG2 and HepG2/CDDP cells were treated with various concentrations of calyxin Y (5, 10, 20 µM) for 24 h. The expression of eEF2K, p-eEF2 and eEF2 was measured by western blot. β-Actin served as a loading control (A). The total RNA was isolated and subjected to real-time PCR for eEF2K mRNA expression (B). **(C)** HepG2 and HepG2/CDDP cells were pretreated with proteasome inhibitor MG132 for 1 h and exposed to calyxin Y (20 µM) for 24 h. The expression of eEF2K was measured by western blot. β-Actin served as a loading control. **(D)** HepG2 and HepG2/CDDP cells were transfected with βTrCP siRNA or negative control (NC) siRNA. Next, the cells were treated with 20 μM calyxin Y for 24 h. The expression of βTrCP and eEF2K was measured by western blot. β-Actin served as a loading control. Bars represent means ± S.D. of three independent experiments; *p < 0.05 and **p < 0.01, compared to non-treated control; ^#^p < 0.05, compared to calyxin Y treated alone or calyxin Y treated NC siRNA transfected cells.

To determine whether eEF2K expression was associated with the effects of calyxin Y, HepG2 and HepG2/CDDP cells were treated with various concentration of calyxin Y (5, 10, 20 µM) for 24 h. Western blot analysis showed that calyxin Y reduced eEF2K expression in a dose-dependent manner (Figure 3A). The inhibition of eEF2K is expected to reduce eEF2 phosphorylation [16]. Consistently, we also found that as the concentration of calyxin Y increased, the level of eEF2 phosphorylation decreased. The total eEF2 level correlated well with the level of β-actin in calyxin Y-treated samples. Next, we decided to research the underlying mechanism by which calyxin Y reduced eEF2K expression. To ascertain whether the reduction of eEF2K induced by calyxin Y occurred at the transcriptional level, we analyzed eEF2K mRNA levels. As illustrated in Figure 3B, real-time PCR indicated that eEF2K mRNA levels were not significantly changed in HepG2 and HepG2/CDDP cells after calyxin Y treatment. To assess the influence of calyxin Y on eEF2K degradation, HepG2 and HepG2/CDDP cells were pretreated with a proteasome inhibitor, MG132. The expression of eEF2K was significantly increased in MG132 treated cells, confirming that the acceleration of protein degradation is involved in calyxin Y-induced inhibition of eEF2K (Figure 3C). It is well established that eEF2K is degraded by the ubiquitin-proteasome system via the SCF βTrCP ubiquitin ligase [17]. To certify the mechanism of calyxin Y in eEF2K degradation, all cells were transfected with βTrCP siRNA to block eEF2K degradation. We found that when eEF2K degradation was blocked by βTrCP siRNA, the inhibition of calyxin Y on eEF2K was reversed (Figure 3D). These results showed that calyxin Y decreased the expression of eEF2K via SCF βTrCP-mediated protein degradation in HepG2 and HepG2/CDDP cells.

### Calyxin Y and CDDP induce a synergistic cytotoxic effect in human HCC cells

**Figure 4:**
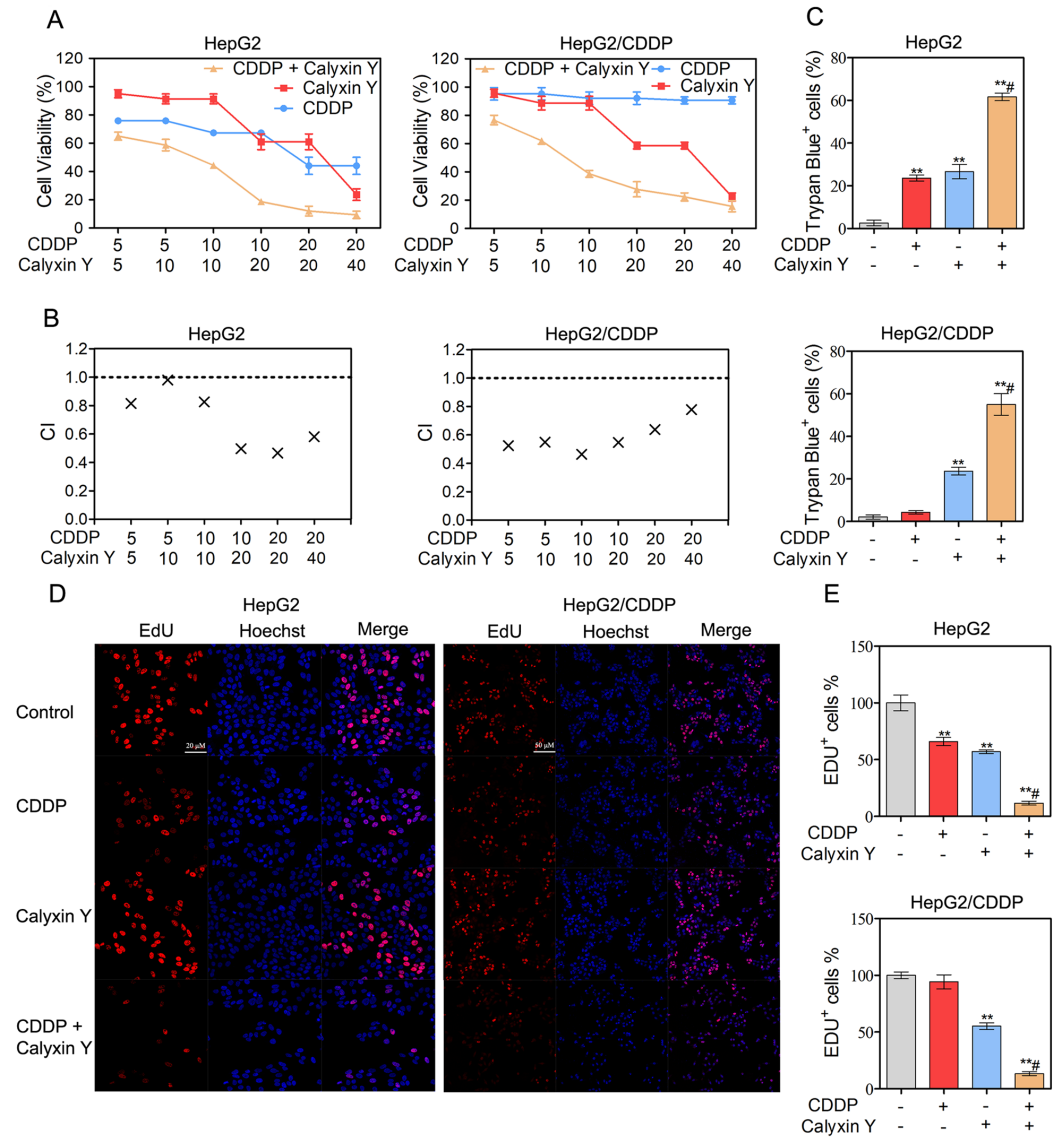
Calyxin Y and CDDP induce a synergistic cytotoxic effect in human HCC cells **(A-B)** HepG2 and HepG2/CDDP cells were treated with the combination of calyxin Y and CDDP at a ratio of 1:1 or 1:2 for 24 h. The cell viability was determined by MTT assay (A). The mean CI values for calyxin Y and CDDP combined treatment were averaged for each experiment and used to calculate the mean between experiments (B). **(C-E)** HepG2 and HepG2/CDDP cells were treated with the combination of calyxin Y (20 µM) and CDDP (10 µM) for 24 h. Unattached and attached cells were collected and stained with trypan blue dye. The percentage of trypan blue^+^ cells represented the population of dead cells (C). The DNA synthesis was investigated with an EdU labeling/detection Kit. EdU-positive cells were marked as red. Hoechst 33342 (blue) was used for nuclear staining. Images were acquired by ImageXpress^®^ Micro Confocal system. The scaled bar represents 50 μm (D). The quantitation of EdU-positive cells was analyzed by Graphpad 5.0 (E). Bars represent means ± S.D. of three independent experiments; *p < 0.05 and **p < 0.01, compared to non-treated control; ^#^p < 0.05, compared to CDDP treated alone.

It has been demonstrated that the inhibition of eEF2K sensitizes cancer cells to chemotherapy [18]. We have demonstrated that calyxin Y inhibited the expression of eEF2K protein levels in HepG2 and HepG2/CDDP cells. Therefore, we attempted to determine whether calyxin Y could enhance the cytotoxic effects of CDDP in HCC cells. MTT data showed that the combination of calyxin Y and CDDP could noticeably inhibit cell growth compared with calyxin Y or CDDP alone (Figure 4A). The analysis of the effects of the drug combination revealed that synergistic effects occurred between calyxin Y and CDDP (Figure 4B). For instance, the minimal combination index (CI) value for the calyxin Y-CDDP was 0.465 in HepG2 cells and 0.462 in HepG2/CDDP cells. The combination of CDDP (10 μM) and calyxin Y (20 μM) was chosen for further study due to its preferable CI in both cells (0.496 in HepG2 cells and 0.547 in HepG2/CDDP cells). In addition, trypan blue exclusion assays also confirmed that calyxin Y and CDDP co-treatment had a greater cell growth inhibition effect than either treatment alone (Figure 4C). The 5-ethynyl-20-deoxyuridine (EdU) incorporation assay was used to further determine the anti-proliferative activity of combined treatment. The number of EdU-positive cells was dramatically decreased after calyxin Y and CDDP co-treatment (Figure 4D and 4E). The results showed that combined calyxin Y and CDDP had a synergistic cytotoxic effect on HepG2 and HepG2/CDDP cells.

### The combination of calyxin Y and CDDP induces cell apoptosis via mitochondrial dysfunction in human HCC cells

**Figure 5:**
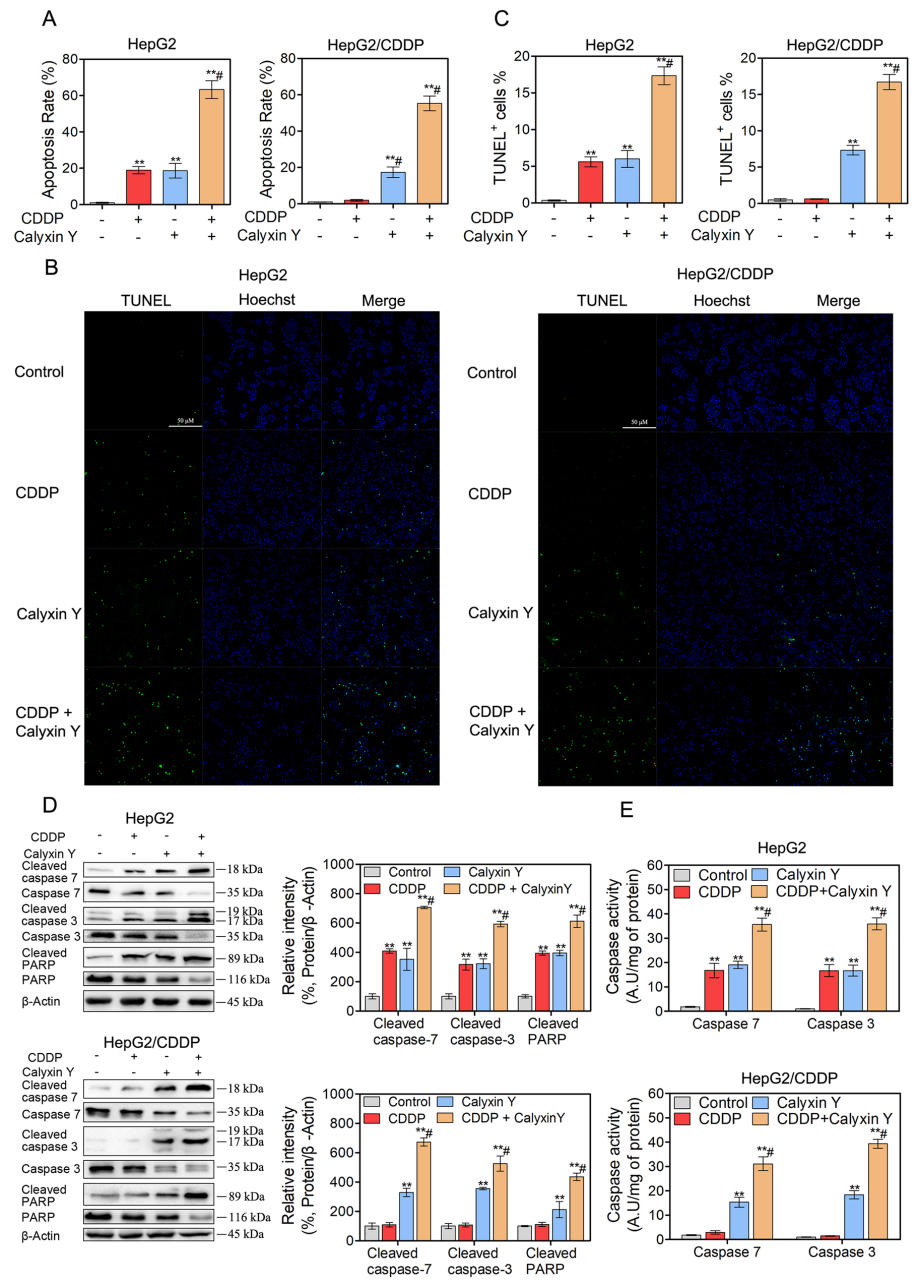
The combination of calyxin Y and CDDP induces cell apoptosis in human HCC cells **(A-E)** HepG2 and HepG2/CDDP cells were treated with the combination of calyxin Y (20 µM) and CDDP (10 µM) for 24 h. The cell apoptosis was assessed by Annexin V/7-AAD assay (A). The DNA fragment was assessed by TUNEL assay. The scaled bar represents 50 µm (B). The quantitation of EdU-positive cells was analyzed by Graphpad 5.0 (C). The expression of cleaved caspase-3/7/PARP and cspase-3/7/PARP was determined by western blot. β-Actin served as a loading control (D). The activity of caspase-3 and caspase-7 was determined by an ELISA reader (E). Bars represent means ± S.D. of three independent experiments; *p < 0.05 and **p < 0.01, compared to non-treated control; ^#^p > 0.05, compared to CDDP treated alone.

**Figure 6:**
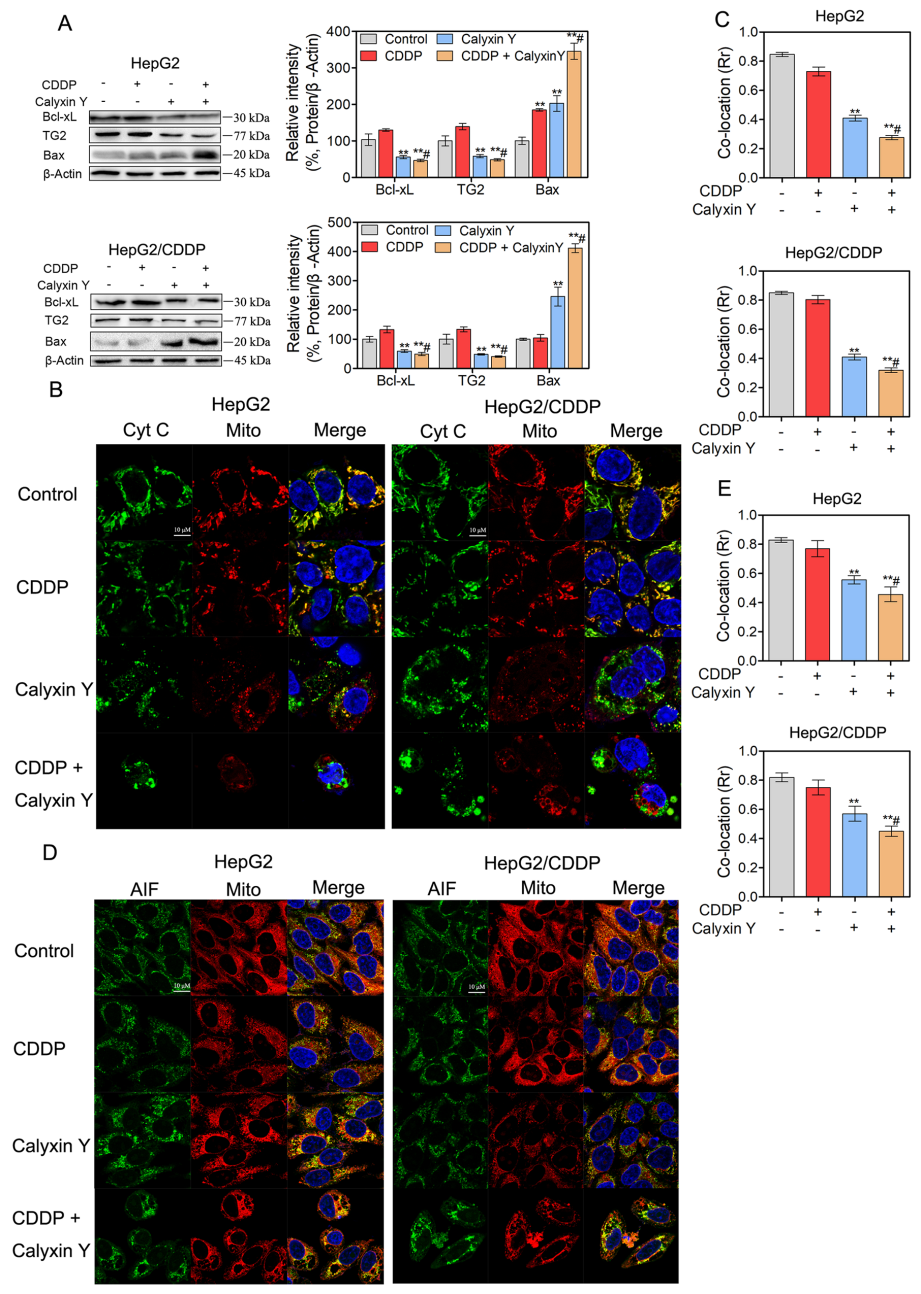
The combination of calyxin Y and CDDP induces mitochondrial dysfunction in human HCC cells **(A-E)** HepG2 and HepG2/CDDP cells were treated with the combination of calyxin Y (20 µM) and CDDP (10 µM) for 24 h. The expression of Bcl-xL, TG2 and Bax was determined by western blot. β-Actin served as a loading control (A). The release of cytochrome c from mitochondria was determined through evaluating the co-localization of cytochrome c (green) and mitochondria-targetable fluorescent probe MitoTracker^®^ Deep Red FM (red) by ImageXpress^®^ Micro Confocal system. The nuclei were stained with Hoechst 33342 (blue). The scaled bar represents 10 µm (B). The quantitation of co-location (Rr) was analyzed by Graphpad 5.0 (C). The release of AIF from mitochondria was determined through evaluating the co-localization of AIF (green) and MitoTracker^®^ Deep Red FM (red) by ImageXpress^®^ Micro Confocal system. The nuclei were stained with Hoechst 33342 (blue). The scaled bar represents 10 µm (D). The quantitation of co-location (Rr) was analyzed by Graphpad 5.0 (E). Bars represent means ± S.D. of three independent experiments; *p < 0.05 and **p < 0.01, compared to non-treated control; ^#^p > 0.05, compared to CDDP treated alone.

To assess the mechanism of the synergistic effects, we hypothesized that calyxin Y would enhance apoptosis in CDDP-treated HepG2 and HepG2/CDDP cells. To test this hypothesis, HepG2 and HepG2/CDDP cells were exposed to CDDP and calyxin Y for 24 h. Apoptosis was measured via flow cytometry with dual staining of Annexin V and 7-Aminoactinomycin D (7-AAD). The results showed that calyxin Y increased CDDP-induced apoptosis in HepG2 cells (from 31% to 63% AV^+^, Figure 5A). Moreover, calyxin Y and CDDP co-treatment led to a marked increase in the number of apoptotic cells compared with calyxin Y alone in HepG2/CDDP cells. Similar effects were observed in the dUTP-digoxigenin nick-end labeling (TUNEL) assay. The results revealed an obvious increase in the number of apoptotic cells after the combination treatment compared with the single agent treatment (Figure 5B and 5C). The executors of apoptosis (cleaved caspase-3, cleaved caspase-7 and cleaved PARP) were measured after co-treatment. As shown in Figure 5D, CDDP alone did not cause the cleavage of caspase-7, caspase-3 or PARP in HepG2/CDDP cells. The combined treatment of calyxin Y and CDDP induced evident cleavage of caspase-7, caspase-3 and PARP in HepG2 and HepG2/CDDP cells. In addition, caspase-7 and caspase-3 activity was also detected. Calyxin Y and CDDP co-treatment resulted in an increase in caspase-7 and caspase-3 activity compared with the single agent treatment (Figure 5E). As a marker of early apoptosis, the decline of mitochondrial membrane permeabilization allows for the release of intermembrane space proteins such as cytochrome c and apoptosis-inducing factor (AIF) to promote caspase-dependent or -independent apoptosis [19]. We then detected the expression of Bcl-2 family proteins (Bcl-xL, tissue transglutaminase (TG2), Bax), which control mitochondrial membrane permeabilization. Bcl-xL and TG2 expression significantly decreased, while Bax expression significantly increased in the combination treatment group compared with the single agent treatment group (Figure 6A). The amount of cytochrome c and AIF in mitochondria after calyxin Y and/or CDDP treatment was assessed via immunofluorescence staining. The decrease of cytochrome c and AIF in mitochondria was detected in calyxin Y and CDDP co-treatment cells (Figure 6B-6E). These data suggested that the combination of calyxin Y and CDDP enhanced caspase-dependent mitochondrial apoptosis compared with a single agent in HepG2 and HepG2/CDDP cells.

### The induction of autophagy contributes to the synergistic cytotoxic effect induced by the combination of calyxin Y and CDDP in human HCC cells

**Figure 7:**
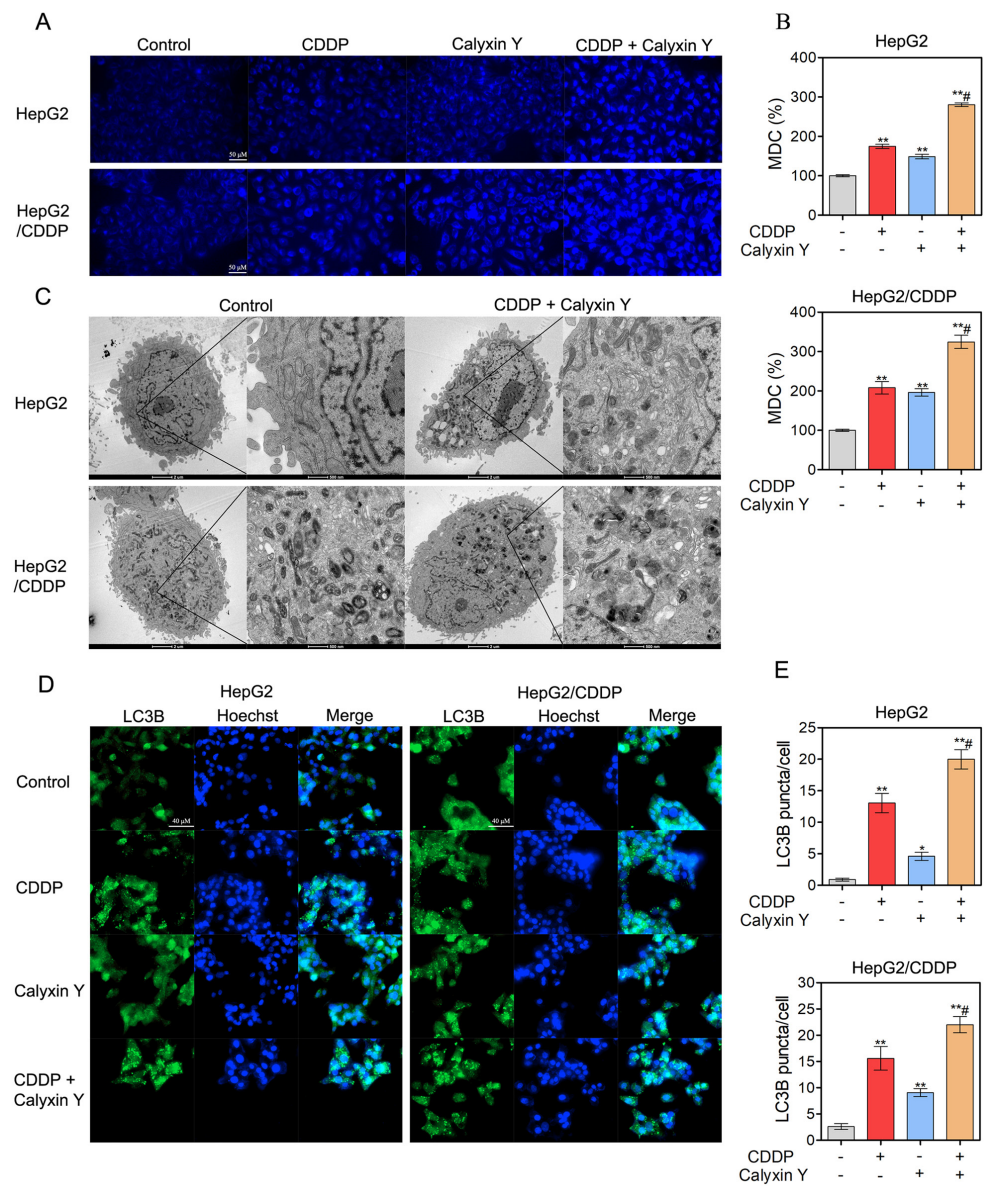
The combination of calyxin Y and CDDP induce the accumulation of autophagosomes in human HCC cells **(A-E)** HepG2 and HepG2/CDDP cells were treated with the combination of calyxin Y (20 µM) and CDDP (10 µM) for 24 h. The cells were loaded with MDC for 45 min. The level of acidic vesicles was determined by confocal microscope. The scaled bar represents 50 µm (A). The quantitation of MDC (%) was analyzed by Graphpad 5.0 (B). The autophagosomes were determined by TEM (C). The generation of autophagosomes was determined by evaluating the puncta of LC3B (green) by ImageXpress^®^ Micro Confocal system. The nuclei were stained with Hoechst 33342 (blue). The scaled bar represents 40 µm (D). The quantitation of LC3B puncta was analyzed by Graphpad 5.0 (E). Bars represent means ± S.D. of three independent experiments; *p < 0.05 and **p < 0.01, compared to non-treated control; ^#^p < 0.05, compared to CDDP treated alone.

**Figure 8:**
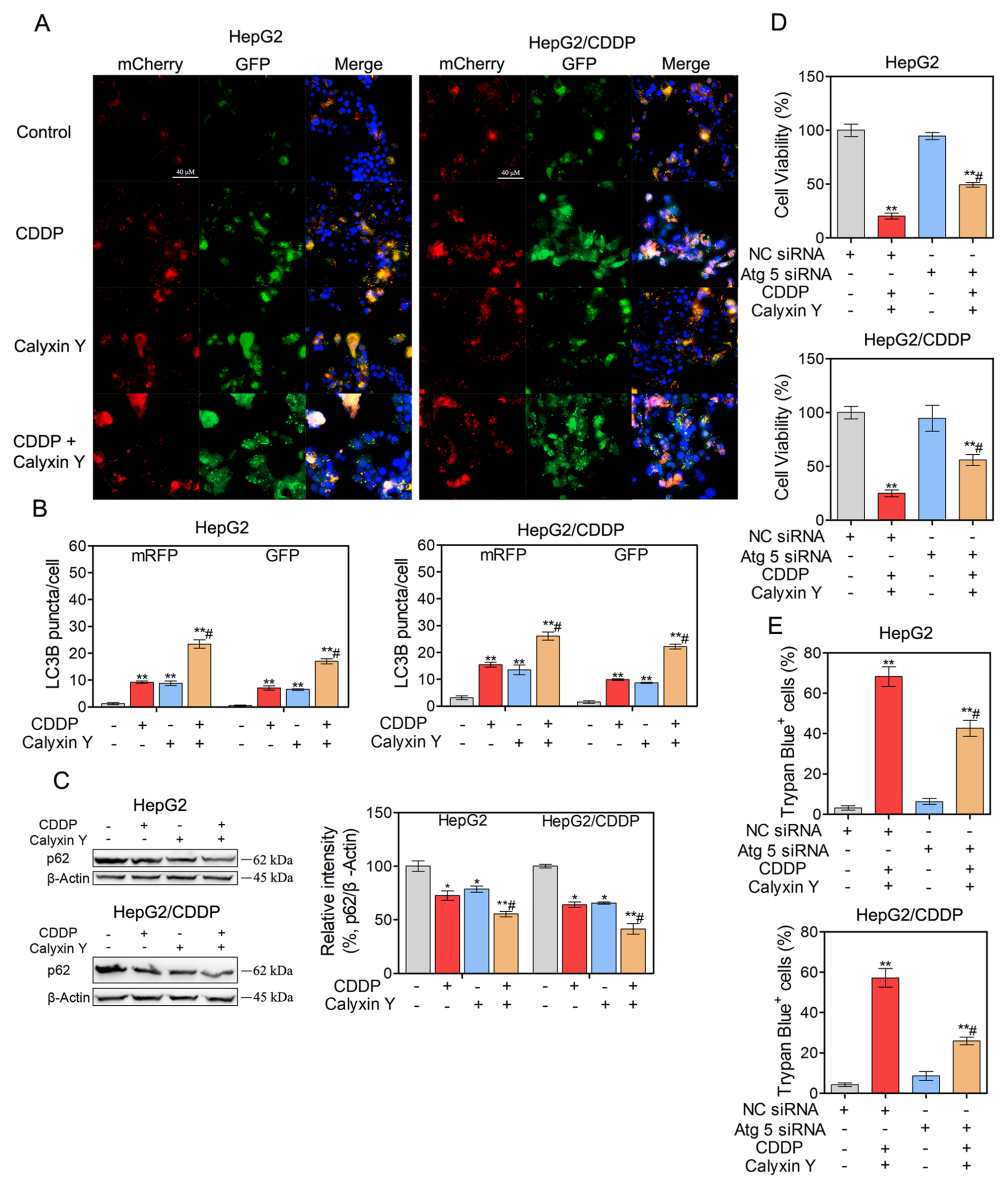
Autophagy contributes to the synergistic effect induced by the combination of calyxin Y and CDDP **(A-B)** HepG2 and HepG2/CDDP cells were transfected with mCherry-GFP-LC3B virus plasmid. After transfection, the cells were treated with the combination of calyxin Y (20 µM) and CDDP (10 µM) for 24 h. The autophagy flux was determined by evaluating the autophagosomes (green and red) and autolysosomes (red) by ImageXpress^®^ Micro Confocal system. The nuclei were stained with Hoechst 33342 (blue). The scaled bar represents 40 µm (A). The quantitation of LC3B puncta was analyzed by Graphpad 5.0 (B). **(C)** HepG2 and HepG2/CDDP cells were treated with the combination of calyxin Y (20 µM) and CDDP (10 µM) for 24 h. The expression of p62 was determined by western blot. β-Actin served as a loading control. **(D-E)** HepG2 and HepG2/CDDP cells were transfected with Atg 5 siRNA or negative control (NC) siRNA. Next, the cells were treated with calyxin Y and CDDP for 24 h. The cell viability was determined by MTT assay (D). Unattached and attached cells were collected and stained with trypan blue dye. The percentage of trypan blue^+^ cells represented the population of dead cells (E). Bars represent means ± S.D. of three independent experiments; *p < 0.05 and **p < 0.01, compared to non-treated control; ^#^p < 0.05, compared to CDDP treated alone or calyxin Y and CDDP co-treated NC siRNA transfected cells.

We next evaluated whether autophagic pathways are involved in the synergistic cytotoxic effect of calyxin Y and CDDP. We used lysosomotropic compound monodansylcadaverine (MDC) to label acidic vesicular organelles, including lysosomes and autophagosomes [20]. Cells co-treated with calyxin Y and CDDP showed a dramatic increase in MDC-labeled vesicles, indicating that co-treatment could induce the formation of acidic vesicular organelles (Figure 7A and 7B). Electron microscopy was further used to monitor autophagosomes, which are double-membraned structures that contain undigested cytoplasmic contents that have not fused with the lysosome; they are characteristic of autophagy [21]. Compared with un-treated cells, a greater number of autophagosomes were observed in calyxin Y and CDDP co-treated HepG2 and HepG2/CDDP cells (Figure 7C). During autophagy, microtubule-associated proteins 1A and 1B (LC3) is required for the formation of autophagosomes [22]; thus, the amount of LC3 positively correlates with the number of autophagosomes. As shown in Figure 7D and 7E, the number of LC3-positive vesicular profiles 0.5-2.0 mm in size was significantly increased in calyxin Y and CDDP co-treated HepG2 and HepG2/CDDP cells. To further evaluate co-treatment induced autophagy either by the increased generation of autophagosomes or the inhibition of autolysosomal degradation, HepG2 and HepG2/CDDP cells were transfected with the mCherry-GFP-LC3B vector system. In this system, autophagosomes are colored yellow, as they express both GFP and mCherry. Autolysosomes are colored red, as they express mCherry alone [23]. Calyxin Y and CDDP co-treatment increased the numbers of both autophagosomes and autolysosomes in HepG2 and HepG2/CDDP cells (Figures 8A and 8B). Consistently, calyxin Y and CDDP co-treatment decreased the level of autophagy-lysosome substrate protein p62 (Figure 8C), indicating the promotion of autophagic degradation. These results suggested that co-treatment induced autophagy by promoting the generation of autophagosomes. We then knocked down Atg 5 to further determine the role of autophagy in the synergistic cytotoxic effects of calyxin Y and CDDP. The MTT and trypan blue exclusion assays showed that Atg-5 siRNA could significantly block cell death induced by calyxin Y and CDDP (Figure 8D and 8E). All these results suggested that the combination of calyxin Y and CDDP induced autophagy, which contributed to the synergistic cytotoxic effect.

### Calyxin Y and CDDP in combination induce cell apoptosis and autophagy via the SCF βTrCP-eEF2K pathway

**Figure 9:**
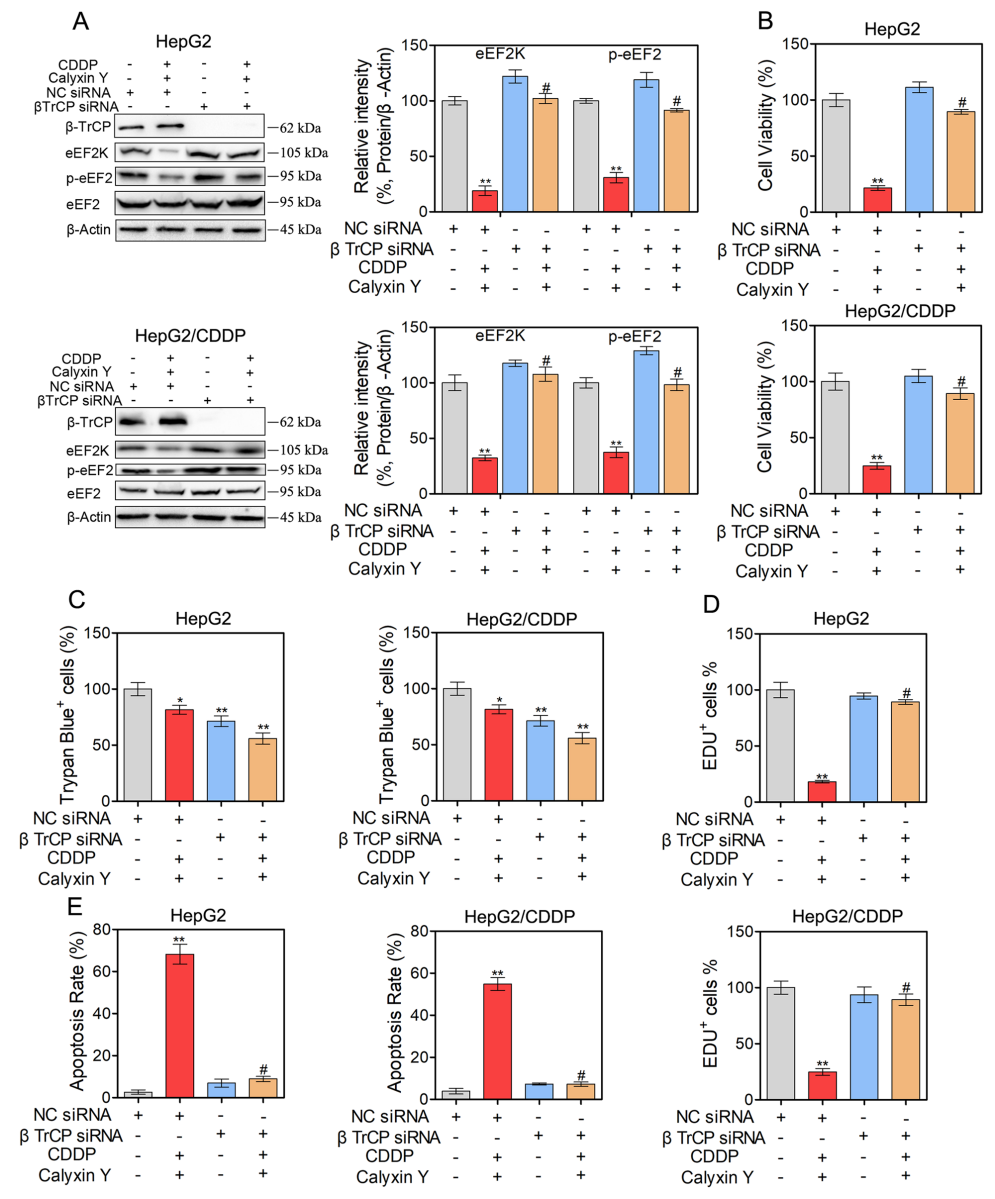
The combination of calyxin Y and CDDP induce cell death involved in SCF βTrCP-eEF2K pathway in human HCC cells **(A-D)** HepG2 and HepG2/CDDP cells were transfected with βTrCP siRNA or negative control (NC) siRNA. Next, the cells were treated with calyxin Y and CDDP for 24 h. The expression of β-TrCP, eEF2K, p-eEF2 and eEF2 was measured by western blot. β-Actin served as a loading control (A). The cell viability was determined by MTT assay (B). Unattached and attached cells were collected and stained with trypan blue dye. The percentage of trypan blue^+^ cells represented the population of dead cells (C). EdU-positive cells were determined by EdU labeling/detection Kit (D). Bars represent means ± SD of three independent experiments; *p < 0.05 and **p < 0.01, compared to non-treated control. ^#^p < 0.05, compared to calyxin Y and CDDP co-treated NC siRNA transfected cells.

**Figure 10:**
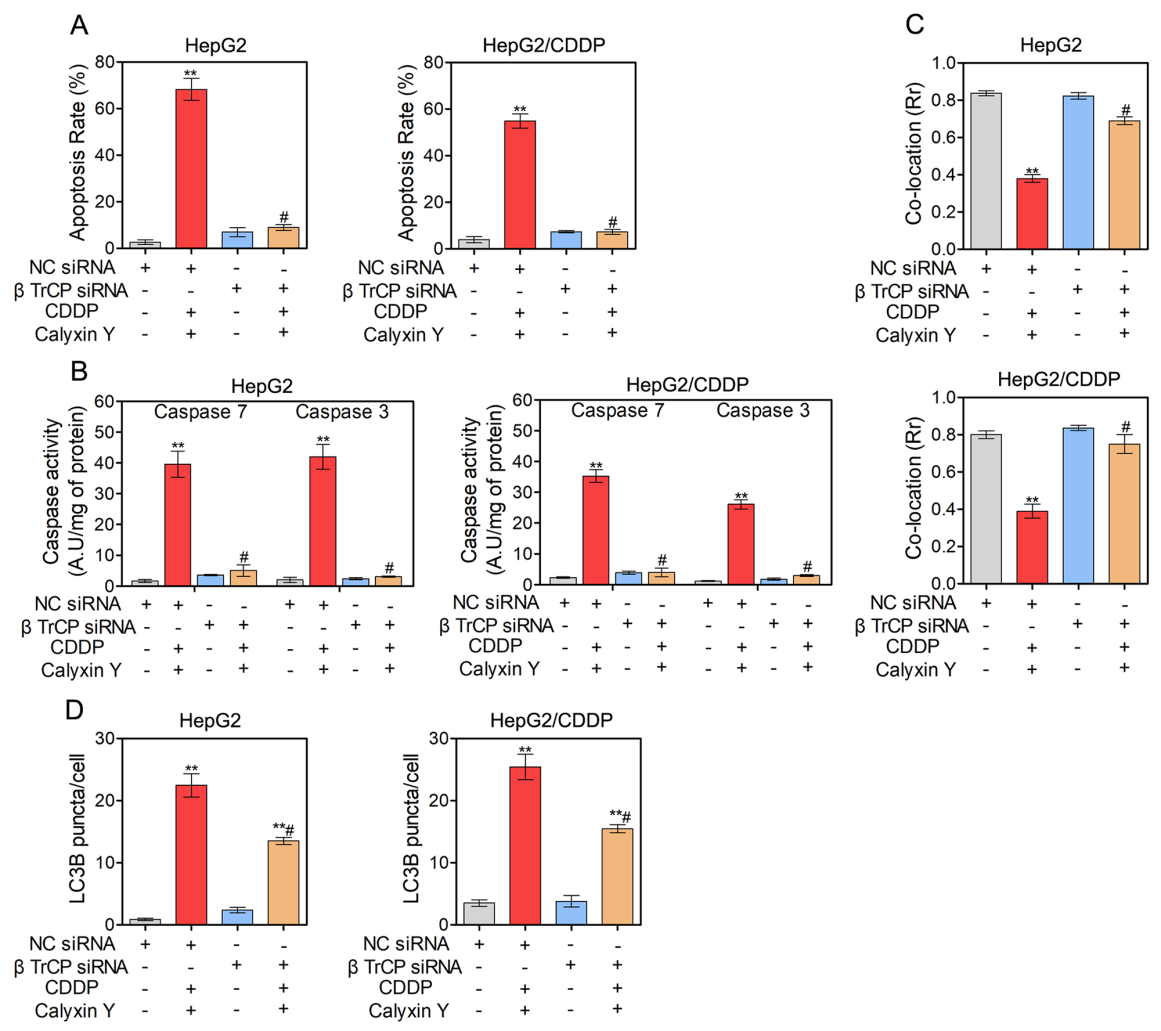
SCF βTrCP-eEF2K pathway contributes to the cell apoptosis and autophagy induced by the combination of calyxin Y and CDDP in human HCC cells **(A-D)** HepG2 and HepG2/CDDP cells were transfected with βTrCP siRNA or negative control (NC) siRNA. Next, the cells were treated with calyxin Y and CDDP for 24 h. The cell apoptosis was assessed by Annexin V/7-AAD assay (A). The activity of caspase-3 and caspase-7 was determined by an ELISA reader (B). The release of cytochrome c from mitochondria was qualified and analyzed by determined by Graphpad 5.0 (C). The quantitation of LC3B puncta was analyzed by Graphpad 5.0 (D). Bars represent means ± SD of three independent experiments; *p < 0.05 and **p < 0.01, compared to non-treated control. ^#^p < 0.05, compared to calyxin Y and CDDP co-treated NC siRNA transfected cells.

We further examined the potential molecular mechanism of the synergistic cytotoxic effect on HepG2 and HepG2/CDDP cells induced by calyxin Y in combination with CDDP. Previous studies have shown that eEF2K is degraded by the ubiquitin-proteasome system via the SCF βTrCP ubiquitin ligase to allow the rapid resumption of translation elongation [17]. We therefore investigated whether the SCF βTrCP-eEF2K pathway is involved in the synergistic cytotoxic effect induced by calyxin Y and CDDP co-treatment. We first knocked down βTrCP by siRNA in HepG2 and HepG2/CDDP cells. In these βTrCP-knocked down cells, the inhibition of eEF2K and eEF2 phosphorylation caused by calyxin Y and CDDP co-treatment was reversed (Figure 9A). As shown in Figure 9B, compared with the control group, silencing of βTrCP completely blocked the inhibition of cell viability induced by calyxin Y and CDDP co-treatment in HepG2 and HepG2/CDDP cells. Trypan blue exclusion assays were used to confirm the role of silencing of βTrCP in the synergistic cytotoxic effect of calyxin Y and CDDP on these cells (Figure 9C). The EdU incorporation assay was used to further validate the effect of SCF βTrCP-eEF2K on the anti-proliferative activity of combined treatment with calyxin Y and CDDP. βTrCP silencing significantly increased the number of EdU-positive cells compared with NC silencing after calyxin Y and CDDP co-treatment (Figure 9D). We further evaluated the involvement of the SCF βTrCP-eEF2K pathway in calyxin Y and CDDP co-treatment induced apoptosis. The result showed that the knockdown of βTrCP markedly attenuated the apoptotic rate induced by the combination treatment both in HepG2 and HepG2/CDDP cells (Figure 10A). Consistently, caspase activity and the release of cytochrome c induced by calyxin Y and CDDP co-treatment were also significantly blocked by βTrCP silencing (Figure 10B and 10C). Moreover, the formation of LC3-positive vesicles induced by calyxin Y and CDDP co-treatment was noticeably inhibited by βTrCP silencing in HepG2 and HepG2/CDDP cells (Figure 10D), indicating the inhibition of autophagy. These results indicated that calyxin Y enhanced CDDP sensitivity was dependent on the SCF βTrCP-eEF2K pathway.

### Calyxin Y and CDDP synergistically inhibit tumor growth in HepG2/CDDP xenograft tumor models *in vivo*

**Figure 11:**
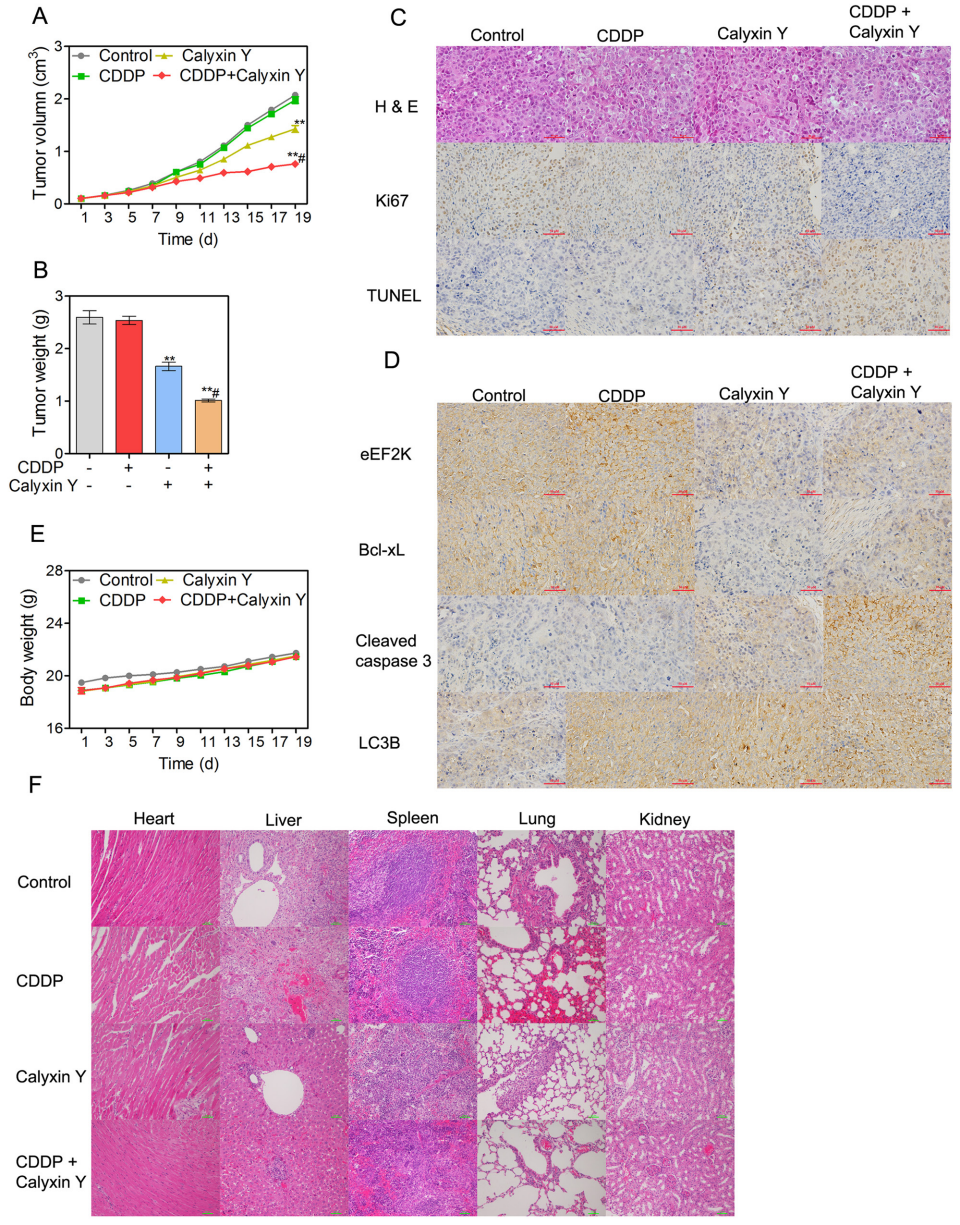
Calyxin Y and CDDP synergistically inhibit tumor growth in HepG2/CDDP xenograft tumor models *in vivo* **(A-F)** BALB/c nu/nu nude mice bearing HepG2/CDDP xenograft model were treated with control (10 % DMEM + 90 % normal saline), calyxin Y (20 mg/kg, i.p. every other day), CDDP (4 mg/kg, i.p. once per week) or the combination of calyxin Y (20 mg/kg, i.p. every other day) and CDDP (4 mg/kg, i.p. once per week) for 19 days (n =6). Tumor volume was recorded every other day (A). Tumor weight was evaluated on the nineteenth day (B). The pathology of tumor specimens was assessed by H&E staining and immunohistochemical staining of Ki67 and TUNEL. The scale bar represents 50 µm (C). eEF2K-mediated apoptosis and autophagy pathway were assessed by immunohistochemical staining of eEF2K, Bcl-xL, cleaved caspase 3 and LC3B. The scale bar represents 50 µm (D). The pathology of heart, liver, spleen, lung and kidney was assessed by H&E staining. The scale bar is 100 µm (E). Heart, liver, spleen, lung and kidney were harvested and stained with H&E (F). The scale bar represents 50 µm. *p < 0.05 and **p < 0.01, compared to non-treated control; ^#^p < 0.05, compared to CDDP treated alone.

To study the effect of the combination of calyxin Y and CDDP *in vivo*, the HepG2/CDDP xenograft model was established. Animals were administered calyxin Y and/or CDDP as described in the Materials and Methods section. Mouse body weight and tumor volume were measured every two days for 19 days. The combination of calyxin Y and CDDP inhibited tumor growth significantly after day 13 compared with the single agent group and the vehicle control group. The combination group had a significantly slower tumor growth rate that resulted in a reduced tumor volume compared with the other three treatment groups (Figure 11A). Mouse tumor weight changes were also used to evaluate the therapeutic effects of drugs. The combination of calyxin Y and CDDP noticeably decreased tumor weight (Figure 11B). The therapeutic efficacy was further evaluated by hematoxylin and eosin (H & E) staining. In the control groups, the tumor cells were tightly aligned and contained large blue-hued nuclei. The tumors in the combination group were characterized by loosely packed cells, which demonstrated the anti-tumor efficacy. Ki67, a marker of cell proliferation, was analyzed via immunohistochemical staining in HepG2/CDDP xenograft tissue. The results revealed that the combination treatment reduced cell proliferation compared with calyxin Y or CDDP-treated mice. The TUNEL assay was used to evaluate the apoptotic cells in tumor tissues. The increased staining intensities revealed the degree of DNA fragmentation induced by the combination of calyxin Y and CDDP (Figure 11C). Immunohistochemistry demonstrated that the combination of calyxin Y and CDDP decreased the expression of eEF2K and Bcl-xL and increased the expression of cleaved caspase-3 and LC3B (Figure 11D), indicating the induction of apoptotic and autophagic cell death *in vivo*. Next, we evaluated the systemic toxicity of the combination of calyxin Y and CDDP *in vivo*. As shown in Figure 11E, all groups demonstrated an increase in body weight over time. In addition, H & E staining of major organs in the control and combination group also did not reveal remarkable abnormalities (Figure 11F). There were no notable changes in the hematological parameters in mice treated with a combination of calyxin Y and CDDP. Moreover, the liver enzyme profiles in the plasma were not changed by the combination treatment (Tables 1 and 2). In short, these results indicated that calyxin Y sensitized liver cancer cells to CDDP, and the combination induced a synergistic antineoplastic effect with a comparative safety profile *in vivo*.

**Table 1:** The hematological parameters of nude mice

Hematological parameters	Control	CDDP	Calyxin Y	CDDP + Calyxin Y
Red blood cells (10^12^/L)	10.8 ± 0.65	9.6 ± 0.67	11.0 ± 0.27	10.6 ± 0.43
White blood cells (10^9^/L)	26.7 ± 0.93	9.9 ± 0.39	15.2 ± 2.2	14.77 ± 1.1
Platelet counts (10^9^/L)	642.5 ± 10.5	562.1 ± 19.2	588.5 ± 80.5	542.5 ± 23.0
Lymphocytes (10^9^/L)	31.8 ± 2.5	8.9 ± 1.9	18.5 ± 1.8	18.6 ± 1.7
Hemoglobin (g/L)	147.2 ± 1.7	126.3 ± 4.7	138.4 ± 7.0	134.7 ± 10.5
Hematocrit (%)	46.8 ± 3.9	44.3 ± 2.8	44.1 ± 1.4	45.3 ± 2.1
Mean corpuscular volume (fL)	45.7 ± 3.1	47.7 ± 3.5	47.3 ± 3.3	44.1 ± 3.1
Mean corpuscular hemoglobin (g/L)	13.9 ± 0.21	13.4 ± 0.29	14.5 ± 0.13	13.8 ± 0.54

Note: Data are means ± S.D., n = 3.

**Table 2:** The hematological biochemical parameters of nude mice

Hematological biochemical parameters	Control	CDDP	Calyxin Y	CDDP + Calyxin Y
Aspartate aminotransferase (U/L)	101.1 ± 2.9	158.5 ± 5.1	129.3 ± 3.5	160.3 ± 4.7
Alanine aminotransferase (U/L)	36.5 ± 3.6	40.2 ± 1.2	37.5 ± 1.4	42.7 ± 1.5
Alkaline phosphatase (U/L)	117.2 ± 4.5	125.4 ± 0.91	115.4 ± 2.7	127.6 ± 3.8

Note: Data are means ± S.D., n = 3.

## DISCUSSION

In the present study, we found that calyxin Y enhanced CDDP-induced cytotoxicity in CDDP-sensitive (HepG2) and resistant (HepG2/CDDP) HCC cells. Also, calyxin Y and CDDP synergistically inhibited tumor growth without causing apparent toxicity effect in HepG2/CDDP xenograft tumor models *in vivo*. Furthermore, calyxin Y and CDDP in combination induced cell apoptosis and autophagy via the SCF βTrCP-eEF2K pathway, which contributed to the inhibition of cell proliferation.

CDDP has been used as a first-line therapy for several solid tumors, including HCC. The antitumor activity of CDDP is believed to be due to its cytotoxicity to cancer cells by damaging DNA and inhibiting DNA synthesis [24]. How cells respond to CDDP-induced DNA damage plays a critical role in CDDP sensitivity. eEF2K plays a key role in the response to DNA damaging stress and has been reported to regulate the sensitivity of cancer cells to several therapeutic drugs, including 2-DG, velcade, curcumin, TRAIL and temozolomide [9, 11-13]. Due to the above results, we evaluated whether eEF2K is associated with CDDP resistance in HCC. Consistent with previous studies, this study showed that silencing eEF2K increased the cytotoxicity of CDDP. Moreover, we found that the natural product calyxin Y, which reduced eEF2K expression in a dose-dependent manner, also increased CDDP-mediated cytotoxicity.

Next, we examined how calyxin Y reduced eEF2K expression. The molecular mechanism of the regulation of eEF2K expression is mainly through transcription control and the ubiquitin-proteasome system. eEF2K expression has been reported to be transcriptionally regulated by Forkhead Box M1 (FOXM1), which is the first transcription factor that directly binds to the promoter region of eEF2K [25]. Moreover, miR-603 can also regulate eEF2K mRNA expression by directly binding to the 3’-UTR regions of eEF2K [26]. In addition to the transcription level of regulation, eEF2K abundance is controlled by the ubiquitin-proteasome system via the SCF βTrCP ubiquitin ligase. eEF2K can be phosphorylated at the Ser-440/Ser-444 degron residues, which in turn was necessary and sufficient for eEF2K to bind to SCF βTrCP [17]. In this study, we found that calyxin Y did not reduce eEF2K expression at a transcriptional level. However, the inhibition of eEF2K by calyxin Y was reversed by the proteases inhibitor MG-132 and βTrCP silencing, indicating that calyxin Y reduced eEF2K expression through SCF βTrCP ubiquitin ligase-mediated degradation.

Resistance to apoptosis, one of the major hallmarks of cancer, is responsible for the poor response of HCC to standard treatment [27]. Mitochondrial apoptosis, which is induced by DNA damage and cellular stress, is inhibited by the anti-apoptotic Bcl-2 protein Bcl-xL. Bcl-xL is thought to decrease the sensitization of cancer cells to CDDP by stabilizing the Δ*Ψ*m and preventing the release of apoptosis-inducing molecules such as cytochrome c, Smac and AIF [28]. In addition, TG2, a Bcl-2 homology 3 (BH3) enzyme involved in protein cross-linking and overexpressed in cancer, has anti-apoptotic effects in cancer cells and plays a key role in the response to CDDP [29]. Our study showed that the combination of calyxin Y and CDDP enhanced mitochondrial apoptosis compared with a single agent, as evidenced by the decreased expression of Bcl-xL and TG2, the increased expression of Bax, and the release of cytochrome c and AIF from mitochondria both in HepG2 and HepG2/CDDP cells. The increased activity of eEF2K has been reported to resist the signal from the apoptosis pathway. The effect of eEF2K on TRAIL-induced apoptosis might be mediated via the modulation of Bcl-xL expression [11]. The over-expression of Bcl-xL can abrogate the sensitizing effect of eEF2K-targeted siRNA on TRAIL-induced apoptosis. Inhibition of the eEF2K/TG2 axis results in caspase-independent apoptosis that is associated with the induction of AIF [30]. Calyxin Y inhibited the expression of eEF2K via βTrCP-mediated degradation. We further evaluated the role of eEF2K in enhanced apoptosis with the combination of calyxin Y and CDDP. Silencing of βTrCP markedly attenuated the apoptotic rate and caspase activity as well as the release of cytochrome c induced by the combination treatment. These results indicated that the inhibition of eEF2K by calyxin Y could enhance the sensitization of CDDP through mitochondrial apoptosis in chemotherapy-sensitive and resistant HCC cells.

Autophagy is a highly conserved process by which cytoplasmic components are sequestered in double membrane vesicles called autophagosomes and are degraded upon fusion with lysosomal compartments [31]. It has been shown that autophagy is primarily a response to the stress of irradiation and serum starvation, especially to DNA stress induced by chemotherapeutic agents [32]. Recent studies reported that silencing of eEF2K actually promoted autophagy, indicating that there is a negative link between eEF2K and autophagy [33]. Consistently, we found that calyxin Y down-regulated eEF2K and induced autophagy. The combination of calyxin Y and CDDP increased autophagosome accumulation and enhanced autophagic flux compared to a single treatment in HepG2 and HepG2/CDDP cells. Autophagy can enhance or inhibit the response of cancer cells to chemotherapy, depending on the particular experimental model. The upregulation of autophagy is correlated with CDDP resistance in many cancers, including lung cancer [34], ovarian cancer [35] and glioma cancer [36]. It has been reported that the pharmacological inhibition of an autophagic response or knockdown of Atg 5 sensitizes CDDP-resistant cells to CDDP [36]. In contrast, Ganoderma tsugae, livin silencing or DIRAS3 overexpression induces autophagic cell death, which increases the chemotherapeutic sensitivity of urothelial cancer, renal carcinoma or ovarian cancer cells, respectively, to CDDP [37-39]. Here, we have demonstrated that autophagy observed in calyxin Y and CDDP co-treated HepG2 and HepG2/CDDP cells represented a mechanism for cell death, indicating a switch from protective autophagy to autophagic cell death. In addition, autophagy induced by calyxin Y and CDDP co-treatment was noticeably inhibited in βTrCP^-^silenced HepG2 and HepG2/CDDP cells. Thus, we suggested that calyxin Y enhanced the level of autophagy through the SCF βTrCP/eEF2K pathway and enhanced the sensitization of CDDP by inducing autophagic cell death in chemotherapy-sensitive and resistant HCC cells.

Taken together, our findings demonstrated that silencing eEF2K enhanced CDDP-induced cytotoxicity in CDDP-sensitive and resistant HCC cells. Furthermore, calyxin Y down-regulated eEF2K by promoting SCF βTrCP-mediated protein degradation and synergistically enhanced the cytotoxic activity of CDDP in HCC cells. Subsequently, we identified a potential mechanism for this cooperative interaction by showing that the combination of calyxin Y and CDDP enhanced apoptotic and autophagic cell death via the SCF βTrCP-eEF2K pathway. Thus, the combined treatment might represent an attractive therapeutic strategy for the treatment of chemotherapy-sensitive and resistant HCC.

## MATERIALS AND METHODS

### Materials and regents

Calyxin Y was isolated from *A. katsumadai* as described before [14]; CDDP was purchased from Sigma-Aldrich (St. Louis, MO, USA), and each had a purity of > 99%. Both compounds were dissolved in DMSO at a stock concentration of 50 mM, and were stored at -20 °C. Cells were treated with DMSO as a control. MDC and 3-(4,5-dimethylthiazol-2-yl)-2,5-diphenyltetrazolium bromide (MTT) were purchased from Sigma-Aldrich (St. Louis, MO, USA). 7-AAD was purchased from Yeasen Biotechnology (Shanghai, China). Dulbecco’s Modified Eagle’s Medium (DMEM) and fetal bovine serum were obtained from Thermo Fisher Scientific (Fair Lawn, NJ, USA). Primary antibodies against eEF2k, eEF2, phospho-eEF2 (Thr56), β-TrCP (D13F10), cleaved caspase-3 (Asp175), caspase-3, cleaved caspase-7 (Asp198), caspase-7, cleaved PARP (Asp214), PARP, Bcl-xL ((54H6), Bax (D2E11), AIF (D39D2), cytochrome c (6H2.B4), p62 (D5E2), β-Actin (13E5); and anti-rabbit IgG and HRP-linked antibodies and anti-mouse IgG and HRP-linked antibodies were purchased from Cell Signaling Technology (Beverly, MA, USA). The TG2 antibody was purchased from Abcam (Cambridge, MA, USA). Lipofectamine 2000 was purchased from Invitrogen (Carlsbad, CA, USA).

### Cell culture

Human hepatocellular carcinoma HepG2 cells were purchased from Cell Bank of Shanghai Institute of Biochemistry and Cell Biology, Chinese Academy of Sciences (Shanghai, China). mtrDNA sequence analysis was done by the cell bank to confirm the species and cells were tested free from mycoplasma. CDDP-selected drug-resistant HepG2/CDDP cells were derived from HepG2 cells by utilizing serial passage in the presence of increasing CDDP concentrations. Briefly, cells were treated with CDDP (1 μM) for 72 h. The media and dead cells were removed, and cells were allowed to recover for a further 72 h and then were treated with a higher concentration of CDDP. This development period was carried out for approximately 6 months, and finally, we obtained the HepG2/CDDP cells. HepG2/CDDP cells were then continuously maintained in the presence of 20 μM CDDP for a further 3 months to maintain stability. All cells were cultured in DMEM media containing 10% fetal bovine serum and incubated with 100 U/ml penicillin and 100 μg/ml streptomycin (Thermo) at 37 °C under an atmosphere of 95% air and 5% CO_2_.

### Cell viability assay

HepG2 and HepG2/CDDP cells were plated in 96-well plates at a density of 5000 cells in 200 μl medium per well and incubated overnight. The cells were treated with calyxin Y and/or CDDP for 24 h, 48 or 72 h. The cell viability of HepG2 and HepG2/CDDP cells was measured by MTT assay as described previously [15].

### Combination index analysis of drug interactions

HepG2 and HepG2/CDDP cells were treated with different concentrations of calyxin Y or CDDP or a combination of the two compounds. Cell viability was examined via the MTT assay. To calculate a CI, computer software CompuSyn (Biosoft, Oxford, UK) was used, taking the entire shape of the cell viability curve into account to calculate whether a combination was synergistic (CI < 0.9), additive (CI = 0.9 - 1.1), or antagonistic (CI > 1.1) [40].

### Trypan blue dye exclusion assay

HepG2 and HepG2/CDDP cells were plated in 96-well plates at a density of 5000 cells in 200 μl of medium per well and were incubated overnight. The cells were treated with calyxin Y and/or CDDP for 24, 48, and 72 h. After treatment, 1000 cells were harvested, and the proportion of dead cells was determined with a hemocytometer (Countstar, Runyu Biotechnology, Shanghai, China); the number of cells stained with trypan blue (Beyotime Institute of Biotechnology, Jiangsu, China) was determined. Trypan blue dye can be excluded from living cells but is able to penetrate dead cells. The dead cells were calculated as follows: trypan blue^+^ cell ratio (%) = (stained cell number/total cell number) x 100.

### 5-ethynyl-20-deoxyuridine (EdU) assay

The DNA synthesis activity of HepG2 and HepG2/CDDP cells was investigated with an EdU labeling/detection Kit (Ribobio, Guangzhou, China) according to the manufacturer’s protocol. In brief, HepG2 and HepG2/CDDP cells were plated on a 96-well plate and incubated overnight. Then, the cells were incubated with calyxin Y and/or CDDP for 20 h. After adding 50 μM EdU labeling agent to the cell culture and incubating for another 8 h, the cells were fixed, permeabilized, and stained with anti-EdU working solution at room temperature. Nuclei were stained with 5 μg/ml Hoechst 33342 (Invitrogen, Carlsbad, CA, USA) for 30 min. Finally, the cells were washed with PBS twice, observed with an ImageXpress^®^ Micro Confocal system (Molecular Devices) and analyzed with MetaXpress software (Molecular Devices).

### DNA fragment detection

To evaluate DNA fragments, a terminal deoxynucleotidyl transferase (TdT)-mediated TUNEL Bright Green Apoptosis Detection Kit (Vazyme Biotech Company, Nanjing, China) was used. HepG2 and HepG2/CDDP cells were plated on 96-well plates and incubated overnight. Next, the cells were treated with calyxin Y and/or CDDP for 24 h. Then, the cells were assessed via the TUNEL assay according to the instructions from the manufacturer. The stained cells were washed with PBS twice, observed with an ImageXpress^®^ Micro Confocal system (Molecular Devices), and analyzed with MetaXpress software (Molecular Devices).

### Annexin V/7-AAD staining assay

HepG2 and HepG2/CDDP cells were plated on 6-well plates and incubated overnight. Next, the cells were treated with calyxin Y and/or CDDP for 24 h. Cells in 200 μl of binding buffer were stained with 5 μl of Annexin V-Alexa Fluor 647 (Fcmacs Biotech Co., Ltd., China) and 5 μl of 7-AAD at room temperature for 15 min. The cells were then analyzed by flow cytometry with a BD Accuri C6 flow cytometer (Becton & Dickinson Company, Franklin Lakes, NJ, USA). The cells undergoing apoptosis are Annexin V positive.

### Caspase activity assay

Caspase activity assay kits (Beyotime Biotech, Nanjing, China) were used to detect caspase activity in accordance with the manufacturer’s protocol. HepG2 and HepG2/CDDP cells were plated on 6-well plates and incubated overnight. Next, the cells were treated with calyxin Y and/or CDDP for 24 h. The cells were lysed, and the supernatant was added to 200 μl of caspase-3 or 7/9 assay reagent for a 1 h incubation. The absorbance was measured at 405 nm with a SpectraMax Plus384 ELISA reader (Molecular Devices). The relative activity was normalized to protein concentrations.

### Transmission electron microscopy (TEM) assay

HepG2 and HepG2/CDDP cells were plated on 6-well plates and incubated overnight. Next, the cells were treated with calyxin Y and/or CDDP for 24 h. The cells were fixed and stained as previously described [16]. The sections were examined with a JEM2100 transmission electron microscope (JEOL, Tokyo, Japan)

### MDC staining assay

HepG2 and HepG2/CDDP cells were plated on 96-well plates and incubated overnight. Next, the cells were treated with calyxin Y and/or CDDP for 24 h. After that, the cells were washed with PBS and stained with 100 mg/ml of MDC in serum-free medium at 37 °C for 15 min. The stained cells were washed with PBS twice and observed with an LSM 700 confocal microscope (Carl Zeiss, Oberkochen, Germany).

### Autophagy flux assay

HepG2 and HepG2/CDDP cells were plated on 96-well plates and reached 60% -70% confluence at the time of transfection. The cells were transfected with Ad-mCherry-GFP-LC3B adenovirus (Hanbio Biotech, Shanghai, China) at an MOI of 30 in 200 µl of DMEM medium containing 10% FBS for 6 h at 37 °C. Following treatment with calyxin Y and/or CDDP for 18 h, the cells were stained with Hoechst 33342 (Yeasen). The stained cells were washed with PBS twice, observed with an ImageXpress^®^ Micro Confocal system (Molecular Devices), and analyzed with MetaXpress software (Molecular Devices).

### Immunofluorescence staining

HepG2 and HepG2/CDDP cells were plated on 96-well plates and incubated overnight. Next, the cells were treated with calyxin Y and/or CDDP for 24 h. Then, the cells were fixed with 4% paraformaldehyde, permeabilized with 0.5% Triton X-100, and blocked with 5% BSA. To detect AIF, cytochrome C and LC3B, the cells were incubated with indicated primary antibodies overnight at 4 °C. Then, the cells were incubated with an Alexa Flour 488-conjugated secondary antibody for 2 h. The mitochondria were stained with MitoTracker^®^ Deep Red FM (Invitrogen). The nuclei were stained with Hoechst 33342 (Yeasen). The stained cells were washed with PBS twice, observed with an ImageXpress^®^ Micro Confocal system (Molecular Devices), and analyzed with MetaXpress software (Molecular Devices).

### Quantitative real-time PCR (qRT-PCR) analysis

Total RNA samples from HepG2 and HepG2/CDDP cells were extracted using RNAiso Plus reagent following the manufacturer’s protocols. RNA (1 μg) was reverse-transcribed using a ReverTra Ace qPCR RT-Kit (Toyobo Life Science, Osaka, Japan) in a MyCycler PCR system (BioRad Laboratories, Hercules, CA). SYBR Green PCR Master Mix was purchased from Toyobo Life Science. The 2^−ΔΔCT^ cycle threshold method was used for the calculation of relative differences in mRNA abundance with a LightCycler 480 qPCR System (Roche Molecular Biochemicals, Mannheim, Germany). Data were normalized to the expression of β-Actin. The results of qRT-PCR were expressed as fold-changes. The normalized value of the target mRNA of the control group is arbitrarily presented as 100. The pairs of primer for PCR were listed below:

eEF2K: (sense) 5′-GGCAAACTCCTTCCACTTCA-3′;
eEF2K: (antisense) 5′-CATCATCCAGCCATTCCC-3′.
β-Actin: (sense) 5′-GCACCACACCTTCTA CAATG-3′;
β-Actin: (antisense) 5′-TGCTTGCTGATCCACATCTG-3′.

### Gene knockdown using small interfering RNA (siRNA)

The siRNAs targeted βTrCP (5′-AAGTGGAATTTGTGGAACATC-3′) [17], eEF2K (5′-AAGCUCGAACCAGAAUGUCAA-3′) [33], Atg 5 (5′-GAGACAAGAA GACAUUAGUdTdT-3′) and non-targeted siRNA was synthesized by Biomics (Nantong, China). HepG2 and HepG2/CDDP cells were transfected with βTrCP siRNA or eEF2K siRNA or Atg 5 siRNA or NC siRNA using Lipofectamine 2000 according to the manufacturer’s instructions. Briefly, the cells were grown in 6-well plates and transfected with 100 nM siRNA for 6 h. Then, cells were assayed by qRT-PCR 24 h after transfection, and the cells in parallel conditions were used for further experiments.

### Western blot analysis

HepG2 and HepG2/CDDP cells were lysed in western blotting lysis buffer and centrifuged at 12000 g for 10 min. The procedures for Western blotting were described previously [41].

### *In vivo* antitumor study

All animal procedures were performed according to comply with the ARRIVE guidelines, the U.K. Animals (Scientific Procedures) Act, 1986 and associated guidelines. The protocol was approved by the Institutional Animal Care and Use Committee at China Pharmaceutical University. Four-week-old BALB/c nu/nu mice (18-22 g) purchased from Shanghai Laboratory Animal Center of Chinese Academy of Sciences (Shanghai, China) were used for *in vivo* experiments. Animals were housed at a constant room temperature with a 12 h:12 h light/dark cycle and were provided a standard rodent diet and water. HepG2/CDDP cells were harvested and injected subcutaneously into the right flank (1 × 10^7^ cells in 150 μl of cold PBS). When tumors reached a volume of 100 mm^3^, the mice were randomly assigned to one of four groups: (1) control group, mice were injected intraperitoneally (i.p.) with 10% DMSO + 90% normal saline (n = 6); (2) calyxin Y group: mice were injected i.p. with 20 mg/kg calyxin Y every other day (n = 6); (3) CDDP group: mice were injected i.p. with 4 mg/kg CDDP once per week (n = 6); (4) calyxin Y + CDDP group: mice were injected i.p. with 20 mg/kg calyxin Y every other day and 4 mg/kg CDDP once per week (n = 6). The tumor volume was determined by measuring length (l) and width (w) and calculating volume (V = 0.5 × l × w^2^) at the indicated time points. At the end of treatment, the animals were sacrificed, and the tumors were removed and weighed for use in histology and protein expression studies. Using a standard saphenous vein blood collection technique, blood was drawn for hematologic and serum biochemistry analyses.

### Histopathology and immunohistochemistry

Formalin-fixed tissue samples were embedded in paraffin and then the paraffin-embedded specimens were cut into serial sections (4-mm thick). Primary tumors, heart, liver, spleen, lung and kidney sections were stained with hematoxylin and eosin, and tumor specimens were immunostained with a Ki-67 (8D5) mouse mAb (1:400, Cell Signaling Technology), an eEF2K antibody (1:500, Abcam), an LC3B (D11) rabbit mAb (1:3200, Cell Signaling Technology), a Bcl-xL (54H6) rabbit mAb (1:300, Cell Signaling Technology) and a cleaved caspase-3 (Asp175) (D3E9) rabbit mAb (1:250, Cell Signaling Technology) at room temperature for 1 h in a humidified chamber. Sections were rinsed with PBS and exposed to appropriate species-specific secondary antibodies for 30 min. Finally, each section was exposed to 3,3’-diaminobenzidine (DAB) solution (KeyGen Biotechnology, China) for 3-5 min after they were rinsed with PBS. To evaluate the apoptotic response in tumor tissues, Colorimetric TUNEL Apoptosis Assay Kits (Beyotime) were used. The sections were deparaffinized, rehydrated through graded alcohols to water, treated with 20 μg/ml proteinase K (37 °C, 20 min) and then washed in 1x Equilibration Buffer. The TUNEL assay was then performed according to the instructions from the manufacturer. Images were captured via microscopy (Carl Zeiss, Germany).

### Statistical analysis

All experiments were performed at least three times unless otherwise stated. The results were analyzed using one-way ANOVA with Tukey multiple comparison test. The data are given as the mean ± standard error (S.D.). *P* value less than 0.05 was considered as significant.

## SUPPLEMENTARY MATERIALS FIGURE



## Abbreviations

CDDP: cisplatin
PARP: poly ADP-ribose polymerase
eEF2K: eukaryotic elongation factor-2 kinase
eEF2: eukaryotic elongation factor-2
SCF: Skp1-Cul1-F-box protein
βTrCP: β-transducin repeat-containing protein
DMSO: dimethyl sulfoxide
7-AAD: 7-Aminoactinomycin D
DMEM: Dulbecco’s Modified Eagle’s Medium
MTT: 3-[4, 5-dimethylthiazole-2-yl]-2, 5-diphenyltetrazolium bromide
EdU: 5-ethynyl-20-deoxyuridine
TUNEL: dUTP-digoxigenin nick-end labeling
AIF: apoptosis-inducing factor
TG2: tissue transglutaminase
TEM: transmission electron microscopy
MDC: monodansylcadaverine
LC3: microtubule-associated proteins 1A and 1B
PCR: quantitative polymerase chain reaction
