# Supplementary material for: Calyxin Y sensitizes cisplatin-sensitive and resistant hepatocellular carcinoma cells to cisplatin through apoptotic and autophagic cell death via SCF βTrCP-mediated eEF2K degradation
Source: Oncotarget. 2017 Aug 3;8(41):70595–616. doi: 10.18632/oncotarget.19883 (PMC5642580; doi:10.18632/oncotarget.19883)
Supplement: Supplementary file 1 [file oncotarget-08-70595-s001.pdf]

Calyxin Y sensitizes cisplatin-sensitive and resistant hepatocellular carcinoma cells to cisplatin through apoptotic and autophagic cell death via SCF βTrCP-mediated eEF2K degradation

SUPPLEMENTARY MATERIALS

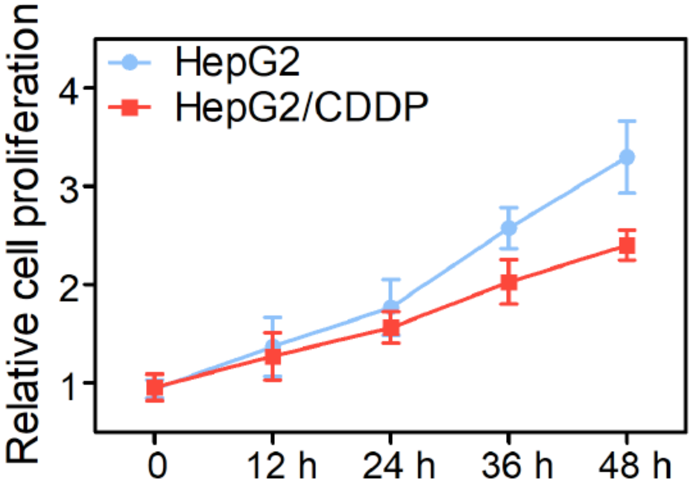

Supplementary Figure 1: The proliferation curve of HepG2 and HepG2/CDDP cells. HepG2 and HepG2/CDDP cells were growing for 12, 24, 36 and 48 h. To count the number of viable cells, Trypan Blue-negative cells were counted using a countess automated cell counter.
